# Modeling the Contribution of Meat to Global Nutrient Availability

**DOI:** 10.3389/fnut.2022.766796

**Published:** 2022-02-02

**Authors:** Nick W. Smith, Andrew J. Fletcher, Jeremy P. Hill, Warren C. McNabb

**Affiliations:** ^1^Riddet Institute, Massey University, Palmerston North, New Zealand; ^2^Sustainable Nutrition Initiative, Riddet Institute, Massey University, Palmerston North, New Zealand; ^3^Fonterra Research and Development Centre, Palmerston North, New Zealand

**Keywords:** sustainable food systems, mathematical modeling, food production, population nutrition, meat

## Abstract

An increasing global population requires increasing food and nutrient availability. Meat is recognized as a nutrient dense food, particularly notable for its high-quality protein content, B vitamin and mineral content. However, it is not known how important meat is currently in nourishing the global population. The DELTA Model was used to calculate the contribution of meat (defined as animal flesh, excluding fish and seafood) to the global availability of 29 nutrients. This model utilizes global food production and use data, coupled with data for food waste, food nutrient composition and nutrient bioavailability to calculate the total amount of each nutrient available for consumption by the global population. Around 333 million tons of meat were produced globally in 2018, 95% of which was available as food, constituting ~7% of total food mass. Meat's contribution to nutrient availability was disproportionately higher than this: meat provided 11% of global food energy availability, 29% of dietary fat and 21% of protein. For the micronutrients, meat provided high proportions of vitamins: A (24%), B1 and B2 (15% each), B5 (10%), B6 (13%), and B12 (56%). Meat also provided high proportions of several trace elements: zinc (19%), selenium (18%), iron (13%), phosphorous (11%), and copper (10%). Meat is a poor contributor to fiber, magnesium and vitamins C and E. Meat was responsible for 16% (cystine) to 32% (lysine) of global availability of the bioavailable indispensable amino acids included in the model, due partly to the high digestibility of these nutrients from meat (83–100%). Of the total meat mass available as food in 2018, 23% was ruminant meat, 34% poultry meat, 32% pig meat, 2% other meat, and 9% offal and fats. The disproportionate contribution of meat to the global availability of nutrients emphasizes its important place in delivering nutrition to the current global population.

## Introduction

Feeding the global population in a sustainable manner is one of the key challenges of the twenty-firsy century (1). The global population is forecast to pass 10 billion by 2060 (2). This has obvious implications for the total quantity of food required; forecasts include a 100% increase in global crop demand between 2005 and 2050 (3), and raising overall food production by 70% in the same period (4). However, there are important nuances to this challenge that must be considered, such as which foods should be targeted for production increases or decreases, and to what extent.

Meat is a dense source of nutrients such as protein, iron, and B vitamins. There are few populations in which meat is not consumed as part of the average diet, and consumption is forecast to increase over the next 10 years, particularly in developing countries (5). However, there exists much criticism in the scientific and popular media of meat consumption from ethical, environmental and health perspectives. It has been proposed that the reduction of global meat production and consumption should be advocated to ensure the sustainability of the global food system and improve human health (6–9). However, there are many facets to this debate and these propositions, particularly those that make blanket recommendations for all global regions, have received criticism from environmental, health, nutrition, and economic standpoints (10–14). Here, results pertaining to the role of meat in human nutrition are discussed, as human nutrition has been recognized by the United Nations as a critical element of sustainability and is key to achieving the Sustainable Development Goals (1).

It is common to discuss the importance of certain nutrients, such as protein, iron, and vitamin A, from a global nutrition perspective. However, while foods rich in these nutrients can be offered as means to increase the supply of these nutrients to at-risk populations in the future, it is less well-known how important certain foods are to the current supply of nutrients. Here, we use a global food system modeling approach to quantify the contribution of meat to the existing supply of essential nutrients.

## Materials and Methods

The results detailed here were obtained from use of the DELTA Model (version 1.3) (15, 16). This model is available online and was developed by the authors as part of the Sustainable Nutrition Initiative.^1^ An overview of the model is given here; a full description can be found in Smith et al. (15).

The DELTA Model is a global food system mass balance model, constructed in R (version 4.0.2). The model uses the Food and Agriculture Organization (FAO) Food Balance Sheets (FBS) as its primary data source (16). Global production and use data (production quantities, feed and food use, supply chain losses and processing chains) from this source for the years 1998–2018 were used to estimate the global availability of food for human consumption in 2018. From this quantity was subtracted inedible portions [according to (17)] and in-home waste fractions [according to (18)], to approximate the total amount of food consumed.

The model then converts this total quantity of food consumed into a total quantity of 29 nutrients consumed (see Table 1 for list of nutrients), by matching food items to nutrient compositions from the United States Department of Agriculture FoodData Central (17). These nutrients were selected based on their presence in nutrient reference value (19) and food composition tables (17), both of which were necessary for their inclusion in the DELTA Model. For protein and the indispensable amino acids (IAA), the model further multiplies the available quantity by a bioavailability coefficient, reflective of the digestibility of these nutrients in the foods from which they are sourced. These coefficients are drawn from (20), and the values for meat are shown in Table 2. Note that seven IAA are included in the DELTA Model; the exclusion of other amino acids is due to limited data availability. Bioavailability of nutrients other than protein and the IAA is not considered, due to insufficient data for many foods, thus the compositional data is unadjusted for the other nutrients.

**Table 1:** Contribution of meat to 2018 global nutrient availability.

	**Nutrient**	**Percentage of**	**Percentage of**	**Percentage of**	**Percentage of**	**Percentage of**	**Percentage of**
		**global availability**	**global availability**	**global availability**	**global availability**	**global availability**	**global availability**
		**provided by**	**provided by**	**provided by**	**provided by**	**provided by**	**provided by**
		**all meat**	**ruminant meat**	**poultry meat**	**pig meat**	**other meat**	**separated offal and fats**
Macronutrients	Carbohydrates	<0.5	<0.5	<0.5	<0.5	<0.5	<0.5
	Energy	11	2	2	4	<0.5	2
	Fat	29	5	6	12	<0.5	5
	Fiber	No contribution	No contribution	No contribution	No contribution	No contribution	No contribution
	Protein	21	6	6	7	1	1
Minerals	Calcium	4	<0.5	3	1	<0.5	<0.5
	Copper	10	1	1	1	<0.5	7
	Iron	13	3	3	2	1	4
	Magnesium	3	1	1	1	<0.5	<0.5
	Phosphorous	11	3	3	4	<0.5	1
	Potassium	7	2	1	2	<0.5	1
	Selenium	18	3	4	9	<0.5	2
	Zinc	19	8	4	5	1	2
Vitamins	Vitamin A	24	<0.5	7	<0.5	<0.5	17
	Vitamin B1 (thiamine)	15	1	1	8	<0.5	1
	Vitamin B2 (riboflavin)	15	3	3	4	<0.5	5
	Vitamin B5 (pantothenic acid)	10	1	4	2	<0.5	3
	Vitamin B6 (pyridoxine)	13	3	4	4	1	1
	Vitamin B9 (folate)	4	<0.5	2	<0.5	<0.5	1
	Vitamin B12 (cobalamin)	56	14	5	5	1	31
	Vitamin C	1	No contribution	1	<0.5	<0.5	<0.5
	Vitamin E	1	<0.5	<0.5	1	<0.5	<0.5
Indispensable amino acids (bioavailability included)	Cystine	16	5	5	5	<0.5	1
	Histidine	26	7	7	9	1	1
	Leucine	21	7	6	7	1	1
	Lysine	32	10	10	10	1	2
	Methionine	25	8	8	7	1	1
	Threonine	25	8	7	8	1	1
	Tryptophan	19	6	5	6	<0.5	1

*Values in the ruminant meat, poultry meat, pig meat, other meat and offal and fats columns may not sum to the value in the all meat column due to rounding*.

**Table 2:** Bioavailability coefficients used for protein and indispensable amino acids in the DELTA Model for meats.

**Nutrient**	**Bioavailability coefficients**	**Bioavailability**
	**for meats (excluding offal)**	**coefficients for offal**
Protein	0.91–1	0.76
Cystine	0.84–1	0.68
Histidine	0.89–0.98	0.7
Leucine	0.89–0.99	0.8
Lysine	0.89–1	0.77
Methionine	0.89–1	0.8
Threonine	0.88–1	0.76
Tryptophan	0.83–1	0.69

Here, values for the contribution of meat (defined as animal flesh, excluding fish and seafood) to the global availability of nutrients were extracted from the model and compared to the global totals for nutrient availability from all foods.

## Results

Approximately 333 million tons of meat commodities left the farm gate globally in 2018 (consisting of 25% ruminant meat, 36% poultry meat, 37% pig meat, and 2% meat from other animals; fish, seafood, eggs, and dairy were not included in this analysis), of which 95% (316 million tons, including offal) was available as food after consideration of non-food and animal feed uses, processing, and supply chain losses. This value constitutes ~7% of total food mass globally, equivalent to roughly 115 grams per person per day in 2018. Table 1 shows the contribution of meat to the global availabilities of the 29 nutrients included in the DELTA Model (version 1.3).

The contribution of meat to nutrition is disproportionately higher than its contribution to global food mass. For the macronutrients, meat is responsible for 29% of dietary fat and 21% of protein. Within that protein, meat supplies between 16% (cystine) and 32% (lysine) of the global availability of the IAA considered by the DELTA Model.

The high contribution of meat to protein and the IAA is partly a result of the high bioavailability coefficients for meat used by the model (Table 2). These range between 0.83 and 1, indicating that a very high proportion of the consumed IAA content can be utilized by the consumer.

The contribution of meat to vitamin availability was most notable for vitamin A and the B vitamins: between 4% [vitamin B9 (folate)] and 56% [vitamin B12 (cobalamin)] of global availability. For the minerals, the largest contributions were to zinc, selenium, iron, phosphorous and copper.

**Figure 1:**
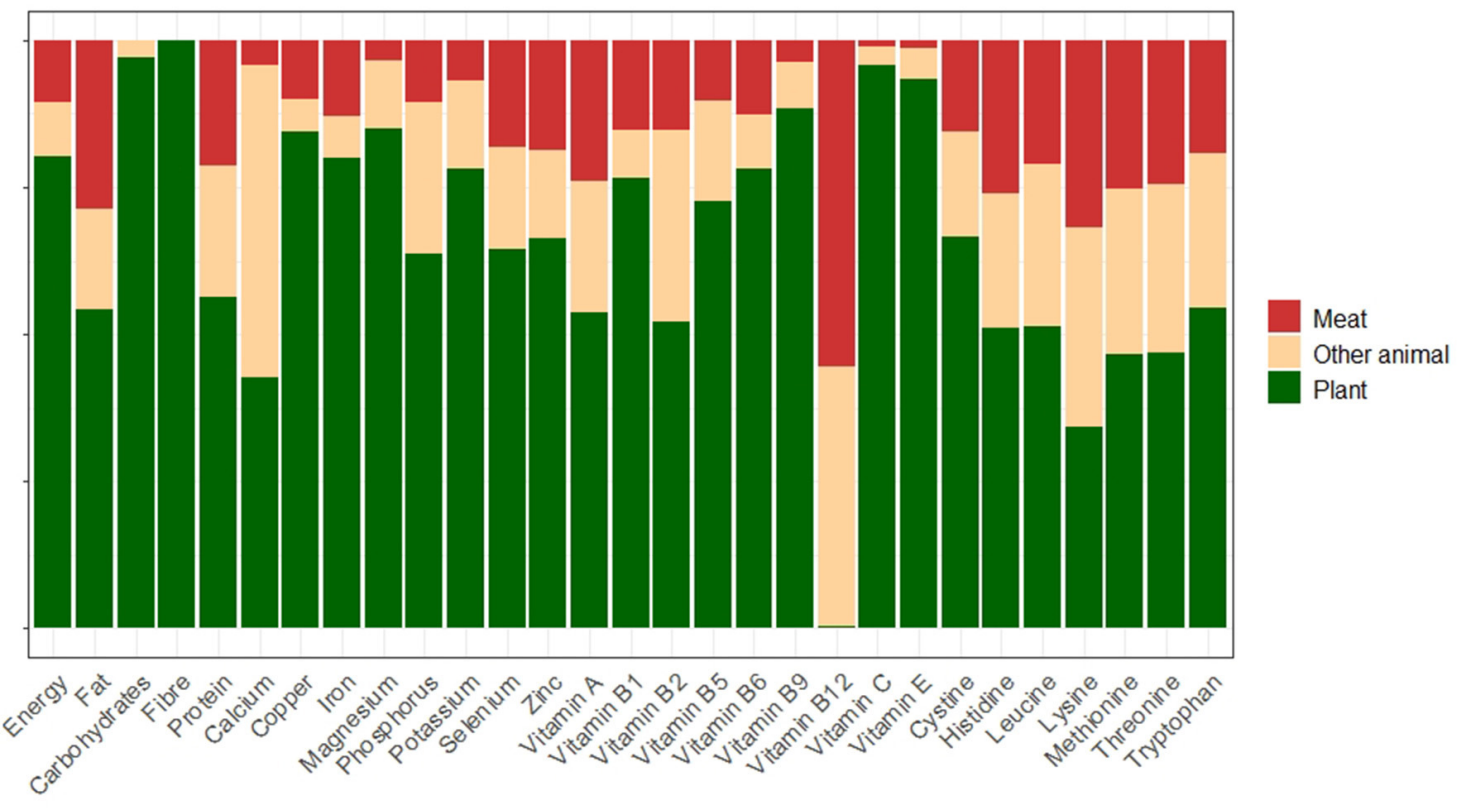
Proportion of the contribution of meat to global nutrient availability compared to other food groups. “Other animal” here refers to all non-meat food products of animal origin: fish and seafood, eggs, and dairy.

On the other hand, meat had very low contributions to the availability of several other nutrients, most notably carbohydrates, fiber and vitamins C and E. This result was expected, as these nutrients are most commonly sourced from plant foods (Figure 1). Indeed, the majority of nutrient supply calculated by the model is plant-sourced, as should be expected given that the model calculates that 75% of global food mass is in this form. Figure 1 illustrates the variability in the main sources of different nutrients and highlights the disproportionate contribution of meat.

**Figure 2:**
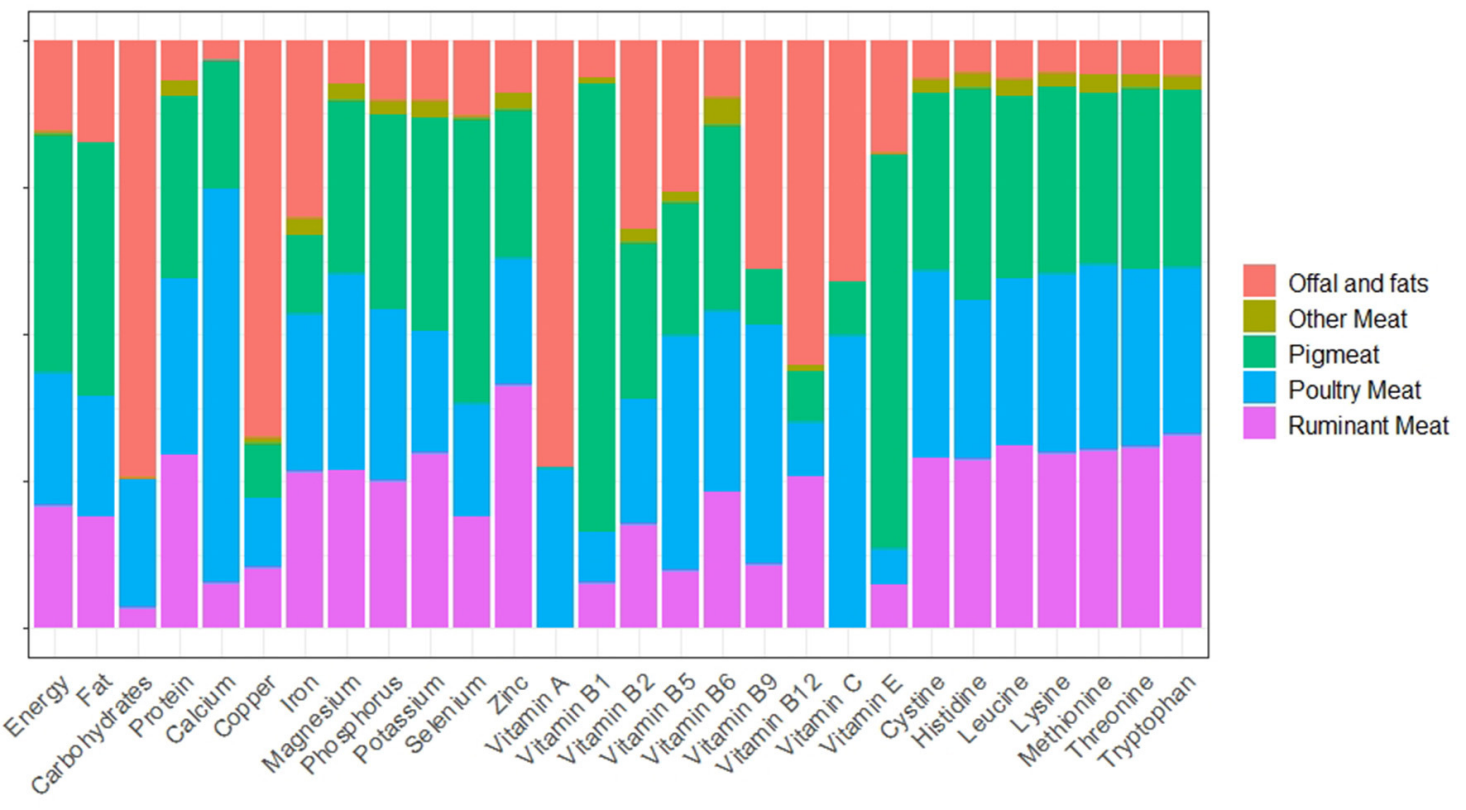
Proportion of the contribution of meat to global nutrient availability supplied by different meat groups.

The DELTA Model results for meat were disaggregated into five groups: ruminant meat, poultry meat, pig meat, other meat, and separated offal and fats. Of the total meat mass available as food (i.e., after losses, in-home waste and non-food uses) in 2018, 23% was ruminant meat, 34% was poultry meat, 32% was pig meat, 2% was other meat, and 9% was separated offal and fats. The contribution of each of these groups to the availability of nutrients from meat varied between nutrients (Figure 2). Offal and fats contributed the majority of copper, vitamin A and vitamin B12; poultry meat was the greatest contributor to calcium; pig meat was the greatest contributor to vitamin B1 (thiamine); and ruminant meat was the greatest contributor to zinc. The availabilities of the IAA and protein were divided almost equally between ruminant, poultry, and pig meat, with minor contributions from other meat and offal and fats.

The in-home waste proportions used by the DELTA Model for meat are sourced from FAO estimates (18). They are between 2 and 11% of the total meat food mass, depending on global region. The inedible portions used by the model for meat [sourced from the (17)] are between 8 and 43% of the total food mass, depending on meat type. For example, poultry meat has a higher inedible portion than ruminant meat due to the lower ratio of meat to bone, skin, etc.

## Discussion

Delivering adequate nutrition to all will be challenging given the forecast population increases and the extent to which malnutrition (both over- and undernutrition) is currently present in the world (21, 22). The challenge is highly complex, with different problems to be tackled in different regional and demographic populations. However, a unifying need is for the adequate supply of all essential nutrients to the entire global population. The DELTA Model demonstrates that global nutrient supply is largely plant-sourced, but with key contributions from animal-sourced foods for many nutrients.

The DELTA Model allows the contribution of individual foods or food groups to global nutrient availability to be quantified, as performed here. The value of this approach is to demonstrate the importance of individual foods in the delivery of specific nutrients. In the case of meat, the DELTA Model shows that meat disproportionally contributes to the availability of many nutrients, in particular protein, fat and several vitamins and minerals. While these results are unsurprising, the DELTA Model provides a novel quantification of global bioavailable nutrient supply.

Limitations of the model include its global perspective. While this gives an overview of total availability of nutrients, it does not capture the variation in availability and affordability in different global regions. Food is not distributed equitably, thus the results presented here do not capture the differing consumption patterns of different populations due to geographic, socio-political and economic factors. This is certainly true of meat: red meat consumption in South Asia and much of Africa is <10 g per person per day on average, compared to 40–70 g in Western Europe, North America, Australasia, and Southern Latin America (23). The model is also limited to evaluating the efficacy of the global food system from a nutritional standpoint, and cannot capture the environmental or economic dynamics of the food system.

Furthermore, bioavailability is only considered for certain nutrients, due to data constraints. The current inclusion of 29 nutrients is also limited for the same reason. The model utilizes food availability data at the level of the FAO FBS, which do not capture the way in which these foods are consumed. For example, the model considers meat at the resolution of the producing animal (e.g., buffalo meat), but not the many food items in which this may be consumed (e.g., steak, burger, sausage, jerky, etc.). Nor does the model consider the impact of the combination of foods into diets (which changes the bioavailability of the nutrients therein), all of which will have an impact on the nutritional value of the food to the individual. The FAO FBS have a number of other limitations, such as omission of certain countries and inconsistent data quality (24), but represent the most complete source of global food data available. Finally, the nutrients considered in this analysis are, in some cases, aggregated groups. For example, the DELTA Model reports availability of vitamin A, but not the relative proportions of retinol and pro-vitamin A within this. Thus, the results of the model are indicative of global nutrient availability and present useful information for the sustainable nutrition debate.

The current importance of meat in supplying bioavailable IAA is clear from the DELTA Model results presented here. The Digestible Indispensable Amino Acid Score (DIAAS) is the current FAO-recommended method for establishing protein quality (25). This score captures the digestibility of IAA in foods, as well as the match between the proportion of IAA in the food compared to bodily requirement. Scores above 1 are considered excellent sources of protein, due to digestibility, complementary ratio compared to bodily requirement and IAA concentration. Beef protein concentrate has a DIAAS of 0.8–1.3, compared to 0.84–0.91 for soy protein isolate, 0.62–0.82 for pea protein concentrate, 0.6 for rice and 0.43 for peanuts (26–29). The combination of the high digestibility of IAA from meat, the high density of these nutrients in meat and the levels of meat consumption globally result in the high contribution of meat to global IAA availability.

A previous publication on the DELTA Model has shown the challenge in meeting global nutrient requirements in the absence of meat (15). Vitamin B12 is a particularly strong example of this, given that more than half of 2018 availability in food was sourced from meat, and total availability only slightly exceeded global requirement (15, 16). Thus, in a no meat scenario for 2030, with all other food production increased by 20%, the DELTA Model predicted a vitamin B12 gap of 35%, alongside gaps for several other nutrients (15). The densest sources of vitamin B12 in the model are meats, fish and seafood, eggs, and dairy, thus a reduction in meat production would have to be matched by increases in the production of these other foods to ensure vitamin B12 sufficiency from food. Alternatives, such as dietary vitamin B12 supplements, were not considered by the model. Simulations with the DELTA Model suggest that a tripling of current dairy production or a quadrupling of current fish and seafood production would be required to meet the vitamin B12 gap through food sources if meat were removed from the system (data not shown^2^). These increases do not appear feasible.

While bioavailability scaling is only included for protein and the IAA in the DELTA Model, research exists on the bioavailability of other nutrients in some foods. In particular, iron and zinc absorption has been shown to be highly dependent on food source and diet, with plant antinutrients such as phytates and oxalates reducing absorption of these and other minerals (30). As such, the World Health Organization (WHO) has recommended increased intakes of these nutrients for diets with high dietary phytate (30). The WHO also noted the higher bioavailability of haem iron from meat, as well as its role in increasing the absorption of non-haem iron. Thus, the role of meat in nutrition extends beyond what is traditionally considered as nutrient content.

Having established the contribution of meat to global nutrition, it is possible to weigh this against the negative health and environmental consequences often connected to meat consumption.

Evidence in the scientific literature linking meat consumption to negative health outcomes varies between meat type and health outcome. The strongest association is between high intakes of processed meat and increased risk of colorectal cancer (31, 32), and the World Cancer Research Fund recommends consuming “no more than moderate amounts of red meat and little, if any, processed meat” for this reason (33). Others have observed links between red and processed meat and specific cancers, metabolic syndrome, and cardiovascular disease in observational studies (34, 35). A meta-analysis of 92 studies of meat intake and health outcomes found 25 studies in which processed meat intake was linked to negative health outcomes, 20 for red meat, and five for total meat (36). They also found two studies in which red meat intake was associated with positive health outcomes, and four studies in low meat consumption populations showing positive outcomes associated with poultry meat intake. There were multiple overlaps between the identified disease states in both the positive and negative associations. These variations in health associations with different meats indicates a need to discuss these associations at the level of individual food items, rather than as an aggregated group. Moreover, individuals with high meat intake tend to have higher BMI, obesity and smoking rates, and lower fruit, vegetable, and whole grain consumption, leading to possible confounding where these factors are not accurately adjusted for (37, 38).

Additionally, the authors of the meta-analysis noted that most of the studies took place in high meat consumption populations in the Americas and Europe, with few in low meat consuming areas in Asia and Africa (36). It is worth noting that these high meat consumption populations are at lower risk of food and macronutrient insecurity than those of developing countries, in which fewer studies on health associations with meat have taken place. The importance and potential of meat to the diet of those with lower incomes is quite different to the diets of wealthier nations: research has shown that small quantities of meat feature in the most affordable nutrient adequate diets for most countries, particularly low- and lower middle income countries (39). It is also these nations that are forecast to increase their demand for meat in coming years (5).

Many national dietary guidelines recommend reduction or constraint of meat intake (40–42). Indeed, the 115 g per person per day available in 2018 from the calculations here exceeds the ~105 g for meat and eggs combined recommended in the US guidelines. There has been criticism of the low strength of scientific evidence to support the stance of these dietary guidelines (10, 43). The Dietary Guideline Recommendations from the NutriRECS consortium advised that adults should continue current levels of red and processed meat consumption, due to low-certainty that diets with reduced quantities of these foods have reduced risk of harmful effects (43). They state that: “the desirable effects (a potential lowered risk for cancer and cardiometabolic outcomes) associated with reducing meat consumption probably do not outweigh the undesirable effects (impact on quality of life, burden of modifying cultural and personal meal preparation and eating habits).” However, others have since criticized the NutriRECS methodology, in particular for the low strength of evidence it gave to observational studies as opposed to long-term interventional studies (which are impractical for nutrition outcomes) thus limiting the possible strength of evidence (44).

Finally, the Global Burden of Disease study estimated that diets high in processed or red meat were attributed <200,000 deaths annually. This is <2% of the number attributed to diets low in specific foods and nutrients: whole grains, nuts and seeds, fruits, vegetables, seafood omega-3 fatty acids, fiber polyunsaturated fatty acids and legumes (23). Global average intakes of processed meat and red meat were found to be 4 and 27 g per person per day in this study, respectively, compared to calculated optimal intakes of 2.1 and 23 g or below. It is the opinion of the authors that remedying excess meat consumption where it reduces consumption of other important foods and nutrients should be the focus of dietary policy. Dietary policy should encourage the consumption of more fruits, vegetables, and dietary fiber, toward a more balanced diet.

Environmentally, agriculture has been estimated to contribute 14.5% of global anthropogenic greenhouse gas emission, 41% of which is from beef production (45). Pig meat and poultry contributed a little under 10% each, together showing the substantial proportion of global agricultural emissions associated with meat production. Concerns have also been raised over the land use, terrestrial acidification, eutrophication, and water use associated with meat production (46). However, large variation was noted between the footprints of different production systems. For example, the global average greenhouse gas emissions for beef reported by these authors were 50 kg CO_2_eq per 100 g protein for beef herd (ranging between 5th percentile value of 19 and 95th percentile of 135), compared with 17 for dairy herd beef (5th percentile: 7.6; 95th percentile: 29) (47). Furthermore, production system plays a role, with some evidence that grass-fed systems have lower emissions than “conventional” systems that use large amounts of concentrate feed (48). These variations for beef are almost certain to exist for all meat production systems and demonstrate the need and potential for improvements in environmental efficiency toward and beyond best practice.

When considering land use, it is important to ascertain land use suitability. Much of the land used by grazed meat animals is not capable of producing human-edible crops; Mottet et al. (49) calculated that, of the two billion hectares of grazing land in 2010, around one third could be converted to cropland, the remainder being unsuitable for this use. However, these authors also report that 560 million hectares of arable cropland (40% of global arable cropland) are currently used to produce animal feed, largely for non-ruminant production. Greater discussion and further research is required to establish what the best use of different land types should be for the delivery of human nutrition and/or for non-food uses.

Finally, consumption of meat is of course a personal choice, influenced by further ethical and social factors not discussed here. Individuals should ensure that their nutrient requirements are adequately met when making this choice. However, the options available to an individual to meet their nutrient requirements are not equivalent to the options available to the global population. The fact that meat can be nutritionally replaced in the diet of an individual does not imply that the same is true for whole populations. This statement can be applied to any food group, emphasizing the need for a systems view of nutrition.

The reasons against meat production and consumption must be weighed against the benefits, foremost among which are the nutritional benefits. It should also be emphasized that meat's contributions to global nutrition reported here come with only 11% of the global availability of energy and only 7% of global food mass, thus proving meat to be nutrient dense from both an energy and mass perspective. Furthermore, whether meat consumption should be increased or decreased is a localized question: Adesogan et al. (12) argue that meat consumption has an essential role in low- and middle-income countries in preventing undernutrition. The Global Burden of Disease study supports this, indicating that several global regions have suboptimal red meat intakes, while others, particularly high-income regions, have excess intakes (23). Inter-population differences within these regions based on access will also exist and require consideration.

The extent to which meat should feature in the human diet is under debate but choice will differ between individuals and populations (11). The DELTA Model demonstrates the current contribution of meat to global nutrition and indicates that a practical replacement, either as a sole or combination of foods, for the full contribution of meat to meeting global nutrient requirements is not currently available and does not appear feasible in the short term (15). The global contribution of meat to human nutrition must be considered in any debate, decision making, or policy on its production and consumption.

## Data Availability Statement

The raw data supporting the conclusions of this article will be made available by the authors, without undue reservation.

## Author Contributions

WM conceived the idea for the manuscript. NS and AF were involved in the construction of DELTA version 1.3, with supervision from JH and WM. NS analyzed the data and wrote the original draft of the manuscript. All authors contributed to reviewing and editing the final version of the manuscript.

## Conflict of Interest

WM sits on an FAO Scientific Advisory Committee for the role of sustainable livestock in the global food system. NS and WM have previously been invited panelists and speakers at events organized by the New Zealand meat industry. NS and WM are employees of Massey University. AF and JH are employees of Fonterra Cooperative Ltd. All authors are affiliated with the Riddet Institute, which has a strategic partnership with Fonterra Cooperative Ltd.

## Publisher's Note

All claims expressed in this article are solely those of the authors and do not necessarily represent those of their affiliated organizations, or those of the publisher, the editors and the reviewers. Any product that may be evaluated in this article, or claim that may be made by its manufacturer, is not guaranteed or endorsed by the publisher.

## Footnotes

^1^ www.sustainablenutritioninitiative.com

^2^These results were simulated with the DELTA Model, available at www.sustainablenutritioninitiative.com.
